# Effect of volume infusion on left atrial strain in acute circulatory failure

**DOI:** 10.1186/s13613-024-01274-6

**Published:** 2024-04-09

**Authors:** Marta Cicetti, François Bagate, Cristina Lapenta, Ségolène Gendreau, Paul Masi, Armand Mekontso Dessap

**Affiliations:** 1 Service de Médecine Intensive Réanimation, AP-HP, Centre Hôpitaux Universitaires Henri Mondor, DHU A-TVB, 1 rue Gustave Eiffel, Créteil Cedex, F-94010 France; 2 Università Cattolica del Sacro Cuore, Roma, Italy; 3 Faculté de Médecine, Groupe de recherche clinique CARMAS, Université Paris Est Créteil, Créteil, F- 94010 France; 4 INSERM U955, Institut Mondor de Recherche Biomédicale, Créteil, F-94010 France

**Keywords:** Acute circulatory failure, Left atrial strain, Fluid responsiveness, Echocardiography

## Abstract

**Background:**

Left atrial strain (LAS) is a measure of atrial wall deformation during cardiac cycle and reflects atrial contribution to cardiovascular performance. Pathophysiological significance of LAS in critically ill patients with hemodynamic instability has never been explored. This study aimed at describing LAS and its variation during volume expansion and to assess the relationship between LAS components and fluid responsiveness.

**Methods:**

This prospective observational study was performed in a French ICU and included patients with acute circulatory failure, for whom the treating physician decided to proceed to volume expansion (rapid infusion of 500 mL of crystalloid solution). Trans-thoracic echocardiography was performed before and after the fluid infusion. LAS analysis was performed offline. Fluid responsiveness was defined as an increase in velocity-time integral (VTI) of left ventricular outflow tract ≥ 10%.

**Results:**

Thirty-eight patients were included in the final analysis. Seventeen (45%) patients were fluid responders. LAS analysis had a good feasibility and reproducibility. Overall, LAS was markedly reduced in all its components, with values of 19 [15 – 32], -9 [-19 – -7] and − 9 [-13 – -5] % for LAS reservoir (LASr), conduit (LAScd) and contraction (LASct), respectively. LASr, LAScd and LASct significantly increased during volume expansion in the entire population. Baseline value of LAS did not predict fluid responsiveness and the changes in LAS and VTI during volume expansion were not significantly correlated.

**Conclusions:**

LAS is severely altered during acute circulatory failure. LAS components significantly increase during fluid administration, but cannot be used to predict or assess fluid responsiveness.

**Supplementary Information:**

The online version contains supplementary material available at 10.1186/s13613-024-01274-6.

## Background

Circulatory shock is present in up to one-third of patients admitted to the ICU and volume expansion represents the first-line therapy [1]. Only 50% of critically ill patients increase their cardiac output after fluid infusion [2], a finding that prompts the accurate investigation of volume status in order to avoid useless fluid administration and deleterious consequences of fluid overload. Hemodynamic assessment of fluid loading should encompass the evaluation of left atrium (LA) performance and its contribution to cardiac function [3].

Left atrium is a complex and dynamic structure that significantly influences cardiovascular performance by actively coordinating with the left ventricle (LV) during the cardiac cycle [4]. LA assumes different roles, functioning as a reservoir during ventricular systole, as a conduit during early ventricular diastole and as a pump that increases ventricular filling during late ventricular diastole [5]. Structural and functional characteristics of LA reflect left ventricular diastolic function, as LA is exposed to LV filling pressure during diastole [6].

Recently, LA phasic function has been studied using speckle-tracking echocardiography [7]. This technique allows analysing and quantifying myocardial deformation by measuring LA strain (LAS) during the three phases of atrial function (reservoir, conduit and contraction). Most of the literature in this area involves cardiology patients. LAS has been shown to predict adverse outcomes in many cardiovascular conditions, namely heart failure [8–12], atrial fibrillation [13–16] and severe valvular defects [17, 18].

Gaps of knowledge in application of LAS in critically ill patients remain significant. The literature on the topic is scarce [19–21] and to date, the pathophysiological meaning of LAS in critically ill patients remains unknown. Few studies conducted in healthy subjects suggest that LAS is influenced by preload variations. LAS reservoir (LASr) is reduced in response to controlled reduction of cardiac loading; this includes tilting manoeuvre [22], Valsalva manoeuvre [23] and continuous positive airway pressure (CPAP) application [24]. Conversely, load alteration during passive leg raising produces an increase in LASr [24]. In patients with renal failure, LASr and LAS conduit (LAScd) were reduced after a preload reduction generated by the haemodialysis session, while LAS contraction (LASct) was unaffected [25, 26]. The effect of a fluid bolus on atrial strain in critically ill patients with acute circulatory failure has never been explored. The primary goal of this study was to describe the change in LAS during volume expansion in patients with acute circulatory failure. As secondary goal, we aimed to test if LAS or LAS variations during volume expansion accurately detect preload responsiveness.

## Methods

### Patients

This prospective observational study was conducted between June 2022 and September 2023 in the medical ICU of Henri-Mondor university hospital, Creteil, France. The study was approved by the ethics committee of the French Society of Intensive Care Medicine (*Société de Réanimation de Langue Française*, SRLF, 23–050). Because we routinely use echocardiography to assess the circulatory status of critically ill patients in our ICU, this technique was considered as a component of standard care and patient’s consent was waived. Written and oral information about the study was given to the patients or families as per French law.

We included adult critically ill patients if the treating physician in charge decided to perform volume expansion because of the presence of acute circulatory failure. Acute circulatory failure was defined by at least one of the following signs: (a) hypotension (systolic arterial pressure ≤ 90 mmHg or decrease of more than 50 mmHg or mean arterial pressure ≤ 65 mmHg); (b) heart rate ≥ 100 bpm; (c) oliguria (diuresis ≤ 0.5 mL/kg/h) for more than 2 h; (d) lactate levels ≥ 2 mmol/L; (e) skin mottling. Exclusion criteria were supraventricular tachyarrhythmia or pacemaker rhythm at the time of inclusion, extracorporeal membrane oxygenation and poor image quality for LA strain analysis. Patients were included according to the availability of investigators and ultrasound systems.

### Data collection

We prospectively collected demographic, clinical and biological information from electronic medical records.

### Echocardiography

Transthoracic echocardiography (TTE) examination was performed by trained clinicians (competent in critical care echocardiography) in the supine position using the cardiac ultrasound probe (1.5–4.5 MHz, M5S-D) of a high-quality ultrasound system (GE Vivid S7 or E9 ultrasound system; GEMS, Buc, France). A standard echocardiographic protocol was used, performing all measurements according to current guidelines [27, 28]. For each patient, a single operator performed all TTE measurements. Echocardiographs were recorded and stored as DICOM files for offline analysis. All measurements were taken at end-expiration. A five-chamber apical view was used to record the velocity-time integral of the flow in the left ventricular outflow tract (LVOT–VTI), by averaging three measurements [29]. Left atrial volumes and left atrial ejection fraction were determined by the application of biplane method.

#### Physiological significance of left atrial strain

Speckle-tracking echocardiography analysis of left atrial deformation allows measurement of LAS and provides information on all phases of atrial function: reservoir, conduit and contraction [28, 30, 31]. Left atrial reservoir phase corresponds to pulmonary venous return during ventricular systole, when LA fills and stretches, generating a positive strain deflection. During the conduit phase, which starts with mitral valve opening and spans early ventricular diastole, LA passive emptying occurs, producing a decrease in LAS corresponding to the first negative deflection in LAS curve. Contraction phase occurs from the onset of atrial contraction and covers late ventricular diastole; it is characterised by atrial wall shortening, generating a second negative deflection on left atrial strain curve [32, 33].

### LAS analysis

**Fig. 1:**
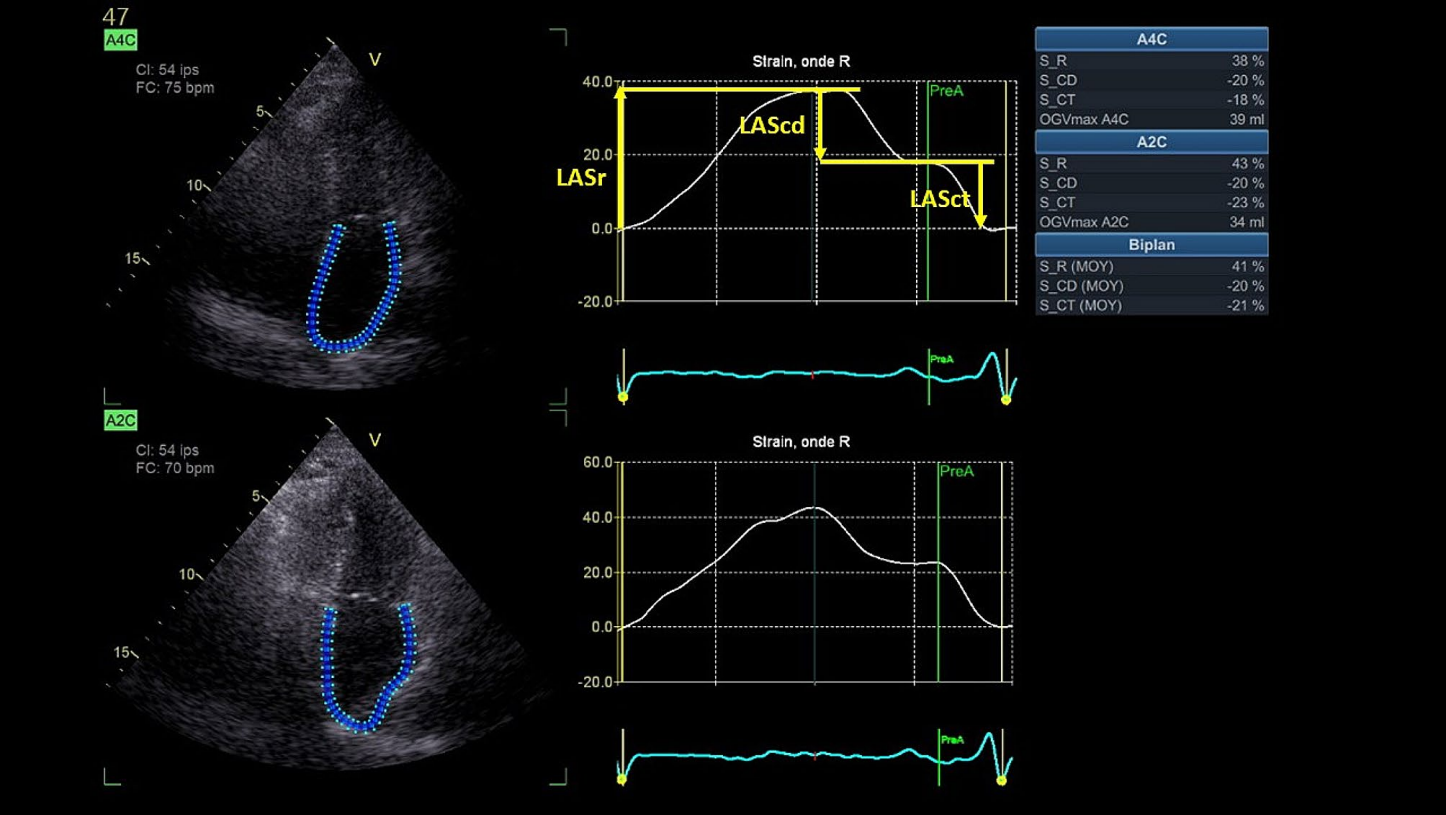
Left atrial strain measurement *A4C indicates apical-four-chamber view; A2C, apical-two-chamber view; LASr, left atrial strain reservoir; LAScd, left atrial strain conduit; LASct left atrial strain contraction, pre-A, pre-atrial contraction

LAS analysis was performed offline (EchoPAC, GE Healthcare) using an automated speckle tracking software with a LAS dedicated mode. LAS included three measurements: LASr, LAScd and LASct, reflecting atrial deformation during different phases of cardiac cycle (Fig. 1). LASr is a positive value while LAScd and LASct are negative values. LAS was measured using optimized apical-four-chamber (A4C) and apical-two-chamber (A2C) views, in order to visualize left atrial endocardium during the entire cardiac cycle. The region of interest (ROI) of the LA was defined by the endocardial border (inner contour of the LA wall) and the epicardial border (outer contour of the LA wall). The regions of interests (ROI) were generated automatically and manual adjustments were performed when necessary. Zero-baseline for obtaining LAS curves was set at end-ventricular diastole using R-R ECG gating. Atrial phases definition and LAS measurements were made in accordance with the European Association of CadioVascular Imaging / American Society of Echocardiography guidelines [30]. We calculated mean LASr, LAScd and LASct by averaging the values recorded in four- and two-chamber views.

The software we used for the analysis of left atrial strain offers and automated measure of tracking quality. Moreover, we visually checked tracking quality by comparing the underlying atrial wall image loop with the superimposed tracking results. Lastly, we analysed the curves derived from that tracking. Only images where the LA wall could be properly traced were included.

### Other measurements

We recorded the following standard hemodynamic data: heart rate, systolic arterial pressure, diastolic arterial pressure, mean arterial pressure, pulse pressure variation, and perfusion index. Respiratory variables, such as respiratory rate, tidal volume and positive end expiratory pressure were also collected.

### Study protocol

At baseline, a first set of hemodynamic and TTE data was collected. Then, a fluid bolus of 500 mL of a crystalloid solution was performed in less than 15 min [34]. A second set of measurements was obtained immediately after volume expansion. Throughout the study period, the ventilator settings and the dose of sedatives and vasoactive drugs were left unchanged. Fluid responsiveness was defined as an increase of 10% or more in LVOT-VTI after volume expansion [35, 36].

### Reproducibility

We assessed intra-observer and inter-observer variability for LAS analysis in a sample of 10 patients that were randomly selected. Inter-observer variability was evaluated by asking two operators (M.C. and F.B.) to perform LAS analysis of the same exams blinded to each other results. Intra-observer variability was evaluated by asking one investigator (M.C.) to repeat LAS analysis with at least 2-week interval, blinded to the results of the first analysis. The repeated analyses were performed on the same pre-selected loops and cardiac cycles. Inter-and intra-observer variability were assessed by intraclass correlation coefficients.

### Statistical analysis

Sample size calculation was based on LASr value. We calculated that a sample size of at least 29 patients would have a 90% power to detect a 5% improvement in LASr after fluid bolus, considering a baseline LASr of 20% with a standard deviation of 9%, based on previous studies evaluating LAS in critically ill patients [19, 21]. Considering the difficulty in obtaining good-quality images in critically ill patients, we planned to enrol at least 40 patients. Data are expressed as mean ± standard deviation or median [interquartile range], as appropriate. Normality of continuous variables was assessed with the Shapiro–Wilk test. Comparisons between before and after fluid administration were assessed through a paired Student’s t test or a Wilcoxon test, as appropriate. Comparisons between fluid responders and fluid non-responders were assessed through a two-sample Student’s t test or a Mann–Whitney U test, as appropriate. Because left atrial strain has been shown to reflect left ventricular filling pressure in patients with heart failure [8, 12], we tested its association with traditional Doppler indices of abnormal diastolic function [37] by using bivariate correlation analysis and summarizing the results in a correlation matrix (corrplot package within the R environment). Correlations were tested using the Spearman method with Benjamini–Hochberg correction to control the false discovery rate at the 0.05 level. Receiver-operating characteristic curves (with 95% confidence interval) were built for the prediction of fluid responsiveness using the baseline value of LAS. Correlations were quantified by the Spearman coefficient. A p-value of less than 0.05 was considered statistically significant.

## Results

### Patient characteristics

**Fig. 2:**
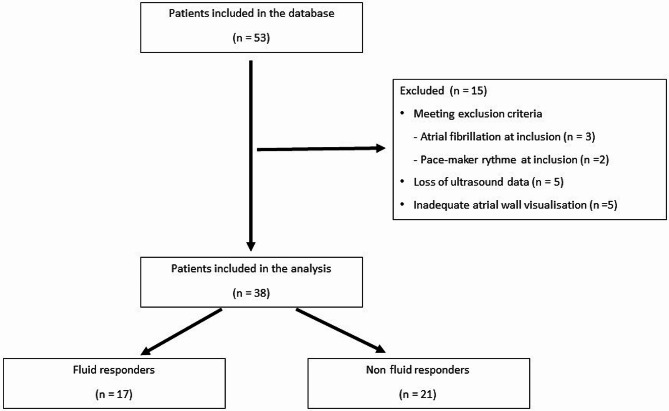
Flow-chart of inclusions

Fifty-three patients were assessed. Ten patients were excluded (including five patients with non-sinus rhythm at the time of inclusion, five patients for whom data were lost for when transferring echocardiographs from ultrasound machine to hard disk), and five patients had inadequate atrial wall visualisation for LAS assessment, leaving 38 patients for analysis (feasibility of 88% [38/43], Fig. 2). All patients were included once.

The baseline characteristics of the studied population are summarised in Table 1. Patients were included a median of 1 [0–2] days after ICU admission (Table 1). At time of inclusion, 21 (55%) patients were receiving noradrenaline infusion, with a median dose of 0.4 [0.2–0.8] mcg/kg/min; 26 (68%) patients were mechanically ventilated with a median value of positive end expiratory pressure of 6 [5–8] cmH_2_O (Table 1). The median ICU length of stay was 13 [5–21] days and the ICU mortality was 21% (Table 1).

**Table 1:** Patients characteristics at baseline

Age - years	59 [43–68]
Sex male – no. (%)	26 (68)
BMI (kg/m^2^)	24.5 [21.7–27.9]
*Comorbidities*	
Hypertension HFrEF HFpEF Paroxysmal atrial fibrillation COPD Diabetes CKD Chronic dialysis Cirrhosis Immunodepression	17 (45%) 5 (13%) 1 (3%) 4 (11%) 2 (5%) 8 (21%) 3 (8%) 2 (5%) 8 (21%) 11 (29%)
*Chronic treatment*	
Beta-blockers ACEIs / ARBs Loop-diuretics Aldactone Amiodarone	11 (29%) 13 (34%) 8 (21%) 3 (8%) 3 (8%)
*Reason for ICU admission*	
Acute respiratory failure Sepsis Shock Septic Cardiogenic Hypovolemic and hemorrhagic Cardiac arrest Neurologic Multidrug intoxication Other	6 (16%) 9 (24%) 3 (8%) 2 (5%) 3 (8%) 4 (11%) 7 (19%) 2 (5%) 2 (5%)
*Severity, supports and outcomes*	
SAPS 2 on admission	47 [35–66]
Days of mechanical ventilation	10 [3–14]
Days on vasopressors / inotropes	3 [2–7]
ICU length of stay, days	13 [5–21]
Hospital length of stay, days	23 [14–37]
ICU mortality - no. (%)	8 (21%)
Hospital mortality - no. (%)	11 (29%)
Days from admission	1 [0–2]
SOFA score	7 [4–12]
*Hemodynamic condition*	
SOFA cardio-vascular score Noradrenaline infusion – no. (%) Noradrenaline dosage – µg/kg/min Dobutamine infusion – no. (%) Dobutamine dosage – µg/kg/min Right ventricular failure – no. (%) Moderate/severe mitral or aortic valvular disease – no. (%)	3 [1–4] 21 (55) 0.4 [0.2–0.8] 2 (5) 2.5 [2.5–2.5] 1 (3%) 1 (3%)
*Respiratory condition*	
Conventional Oxygen Therapy – no. (%) High Flow Nasal Cannula – no. (%) Non Invasive Ventilation – no. (%) Invasive Mechanical Ventilation – no. (%) PEEP – cmH2O FiO2	5 (13%) 3 (8%) 1 (3%) 26 (68%) 6 [5–8] 0.4 [0.3–0.6]
*Biology*	
Lactate – mmol/L [37 pts] Creatinine - µmol/L Haemoglobin – g/dL	1.7 [1.1–4.3] 116 [81–180] 9.7 [8.6–11.1]

* Data are expressed as number (percentage) and median (interquartile range). BMI indicates Body Mass index; HFrEF, Heart Failure reduced Ejection Franction; HFpEF, Heart Failure preserved Ejection Fraction; COPD, chronic obstructive pulmonary disease; CKD, chronic kidney disease; ACEIs, Angiotensine Converting enzyme Inhibitors; ARBs, Angiotensin Receptor Blockers; SAPS2, Simplified Acute Physiology Score; pts, patients; ICU, Intensive Care Unit, SOFA, Sepsis-Related Organ Failure Assessment; PEEP, Positive End Expiratory Pressure; FiO2, Fraction of Inspired Oxygen; pts, patients

### Hemodynamic variables

Overall, fluid loading induced an increase in arterial pressure, cardiac output and pulse oximetry, and a decrease in pulse pressure variation (Table 2). Seventeen (45%) patients were fluid responders. There was no significant difference in hemodynamic characteristics at baseline between fluid responders and non-responders (Table 2).

### Echocardiographic variables

Table 2 displays the echocardiographic parameters at baseline. Mean LAS was feasible in 36 patients. The intra- and inter-observer variability was good for all components of LAS, with intraclass correlation coefficients all above 0.8 (Table S1). All LAS components were markedly reduced (twice lower) at baseline as compared to reported reference values. All LAS components significantly increased during volume expansion (Table 2). Within groups, LASr increased both in fluid responders and non-responders, while LAScd increased only in non-responders and LASct increased only in responders (as also did left ventricle global longitudinal strain) (Table 3, Figure S1-S3). None of the components of LAS at baseline predicted fluid responsiveness (Table 2, Figure S4), and the change in LAS was not associated with the change in LVOT-VTI during fluid loading (Figure S5). The correlations between LAS variation al LV global longitudinal strain variation in responders and non-responders are shown in Figure S6. There was no difference between fluid responders and non-responders concerning parameters of left ventricular and left atrial structure, except for lower values of left ventricle volumes in the former group (Table 2). Indices of diastolic function were similar between groups, apart from higher values of E-wave deceleration time in responders. In the correlation matrix, most indices of increased left ventricular filling pressure were not associated with LAS, nor in the entire cohort nor in non-responder patients. (Figure S7).

**Table 2:** Hemodynamic and echocardiographic data at baseline

	Entire population (*n* = 38)	Fluid responders (*n* = 17)	Non - responders (*n* = 21)	*p*
*Hemodynamic variables*				
HR – bpm SAP – mmHg	98 [80–109] 110 [91–121]	95 [75–103] 110 [91–121]	102 [81–115] 110 [91–120]	0.38 0.62
DAP – mmHg MAP – mmHg	55 [47–60] 73 [64–80]	54 [50–63] 73 [65–80]	55 [45–58] 72 [64–78]	0.66 0.52
PPV – % [24 pts]	10 [7–14]	9 [8–12]	10 [6–17]	0.70
*Echocardiographic variables*				
LVEF – % LVEDV – mL LVESV - mL LVOT-VTI mean – cm CO – L/min [35 pts] GLS-LV – % [32 pts]	54 [44–60] 87 [70–111] 41 [33–54] 21 [15–26] 6.5 [5.7–7.7] -11.3 [-14.5 – -8.1]	54 [51–59] 82 [64–95] 37 [32–47] 20 [16–26] 5.9 [5.3–6.9] -12.6 [-14.1 – -10.6]	54 [43–60] 105 [80–129] 50 [33–62] 22 [15–26] 7.1 [6.1–9.5] -9.5 [-14.6 – -6.6]	0.52 0.04 0.04 0.37 0.01 0.20
E – cm/s [37 pts] DTE – ms A – cm/s [37 pts] E/A [37 pts] E’ lat – cm/s[37 pts] E/E’ lat [37 pts] LA area – cm2 LA volume – mL LAVI – mL/m2 LAEF - % LASr mean – % [36 pts] LAScd mean – % [36 pts] LASct mean – % [36 pts]	70 [61–85] 213 [130–255] 75 [59–90] 0.94 [0.75–1.12] 10 [8–12.9] 7.1 [5.5–9.2] 16 [13–21] 35 [27.25–51.5] 20 [17–26] 54 [41–60] 19 [15–30] -9 [-18 – -7] -9 [-13 – -6]	73 [60–86] 249 [223–267] 77 [58–89] 0.95 [0.78–1.09] 10 [9–12] 7.0 [5.3–9.6] 16 [13–16] 33 [18–42] 20 [10–26] 53 [41–62] 18 [16–32.5] -9.5 [-19 - -7] -8 [-13 – -6]	70 [61–85] 151 [125–235] 73 [60–93] 0.94 [0.74–1.28] 10 [6–13] 7.4 [6.3–9.3] 18 [15–23] 42 [29–53] 20 [16–27] 54 [44–58] 19 [14–31] -9 [-18 – -7] -9 [-14 – -4]	0.72 0.01 0.50 0.96 0.47 0.48 0.07 0.14 0.29 0.83 0.78 0.65 > 0.99

* Data are expressed as number (percentage) or median (interquartile range). HR indicates Heart Rate; bpm, beats per minute; SAP, Systolic Arterial Pressure; DAP, Diastolic Arterial Pressure; MAP, Mean Arterial Pressure; PPV, Pulse Pressure Variation; pts, patients; LVEF, Left ventricular ejection fraction; LVEDV, left ventricular end diastolic volume; LVESV, left ventricular end systolic volume; LVOT-VTI, Left ventricle outflow tract - velocity-time integral; CO, Cardiac Output; GLS-LV, global longitudinal strain – left ventricle; E-wave early diastole; A, A-wave, atrial contraction; DTE, deceleration time E-wave; E’ lat, lateral early diastolic annulus relaxation velocity; LA, left atrium; LAVI, left atrial volume index; LAEF, left atrial ejection fraction; LAS, left atrial strain; LASr, LAS reservoir; LAScd, LAS conduit; LASct, LAS contraction

**Table 3:** Hemodynamic and echocardiographic variables during fluid expansion in responders and non-responders

	Fluid responders (*n* = 17)			Non-responders (*n* = 21)		
	Before fluid bolus	After fluid bolus	p	Before fluid bolus	After fluid bolus	p
HR – bpm SAP – mmHg	95 [75–103] 110 [91–121]	95 [76–104] 120 [111–133]	0.91 < 0.001	102 [81–115] 110 [91–120]	98 [80–110] 115 [101–138]	0.05 0.004
DAP – mmHg	54 [50–63]	60 [54–70]	0.001	55 [45–58]	55 [51–66]	0.006
MAP – mmHg LVEF – % LVEDV – mL LVESV - mL	73 [65–80] 54 [51–59] 82 [64–95] 37 [32–47]]	81 [77–88] 55 [50–60] 89 [76–96] 37 [32–48]	< 0.001 0.84 0.30 0.48	72 [64–78] 54 [43–60] 105 [80–129] 50 [33–62]	74 [68–83] 51 [41–58] 102 [80–120] 48 [40–68]	0.03 0.93 0.56 0.45
LVOT-VTI mean – cm	20 [16–26]	23 [20–29]	< 0.001	22 [15–26]	20 [15–26]	0.69
GLS-LV – % [32 pts] E – cm/s DTE – ms A – cm/s E/A E’ lat – cm/s E/E’ LA area – cm2 LA volume – mL LAVI – mL/m2 LAEF - %	-12.6 [-14.1 – -10.6] 73 [60–86] 249 [223–267] 77 [58–89] 0.95 [0.78–1.09] 10 [9–12] 7.0 [5.3–9.6] 16 [13–16] 33 [18–42] 20 [10–6] 53 [41–62]	-14.6 [-16.9 – -11.6] 80 [69–95] 220 [187–264] 74 [59–91] 1.04 [0.89–1.21] 10 [8–13] 7.4 [5.9–10.1] 18 [15–22] 34 [23–43] 23 [13–28] 60 [52–67]	0.04 < 0.001 0.51 0.08 0.27 0.55 0.07 0.01 0.63 0.64 0.08	-9.5 [-14.6 – -6.6] 70 [85–61] 151 [125–235] 73 [60–93] 0.94 [0.74–1.28] 10 [6–13] 7.4 [6.3–9.3] 18 [15–23] 42 [29–53] 20 [16–27] 54 [44–58]	-13.7 [-17.8 – -8.3] 74 [57–101] 191 [158–238] 78 [60–96] 0.94 [0.75–1.29] 10 [6–13] 8.3 [5.8–11.1] 18 [15–20] 43 [27–58] 19 [13–31] 57 [43–59]	0.13 0.25 0.30 0.97 0.53 0.29 0.37 0.28 0.67 0.62 0.98
LASr mean – % [36 pts]	18 [15–33]	25 [19–34]	0.005	19 [14–31]	25 [18–36]	0.007
LAScd mean – % [36 pts]	-10 [-21 – -6]	-12 [-20 – -9]	0.13	-9[-18 – -7]	-13 [-21 – -8]	0.03
LASct mean – % [36 pts]	-8 [-14 – -6]	-11 [-16 – -8]	0.02	-9 [-14 – -4]	-10 [-16 – -6]	0.40

* Data are expressed as number (percentage) or median (interquartile range). ). HR indicates Heart Rate; bpm, beats per minute; SAP, Systolic Arterial Pressure; DAP, Diastolic Arterial Pressure; MAP, Mean Arterial Pressure; LVEF, Left ventricular ejection fraction; LVEDV, left ventricular end diastolic volume; LVESV, left ventricular end systolic volume; LVOT-VTI, Left ventricle outflow tract - velocity-time integral; CO, Cardiac Output; GLS-LV, global longitudinal strain – left ventricle; E-wave early diastole; A, A-wave, atrial contraction; DTE, deceleration time E-wave; E’ lat, lateral early diastolic annulus relaxation velocity; LA, left atrium; LAVI, left atrial volume index; LAEF, left atrial ejection fraction; LAS, left atrial strain; LASr, LAS reservoir; LAScd, LAS conduit; LASct, LAS contraction

## Discussion

We herein report the first study on LAS during fluid loading in the critically-ill with the following main findings: (i) LAS was feasible with good reproducibility in critically ill patients with circulatory failure; (ii) all three components LAS were severely altered at baseline and markedly increased with fluid administration overall; (iii) the baseline values of LAS did not predict fluid responsiveness, and the change in LAS was not correlated to the change in cardiac ejection.

### LAS in critically ill patients

Several studies have reported normal reference values of LAS derived from large populations of healthy adult subjects [38, 39] as follows: LASr 42 (36–48) %, LAScd − 26 (-20 to -32) %, and LASct − 16 (-13 to -19) %. There are few studies evaluating LAS in ICU patients [19–21], showing variable results. Franchi et al. [20] found normal values of LASr (40.2 ± 12.0) in a cohort of patients undergoing invasive mechanical ventilation, with stable hemodynamic status. On the contrary, Beyls et al. [19] reported that in COVID-19 patients developing atrial fibrillation during ICU stay, LAS parameters were severely reduced: LASr 20.2% [12.3–27.3], LAScd − 8.1% [-6.3 to -10.9], LASct − 9.7% [-5.2 to -16.1]. Cameli et al. [21] also found a profound reduction of LASr in critically ill mechanically ventilated adults with a pulse pressure variation < 15%; however, the investigators did not provide a detailed description of hemodynamic status of the studied population. Our study shows that during circulatory failure, all three components of LAS are severely altered: LASr 19% [15–32], LAScd − 9% [-19 to -7] and LASct − 9% [-13 to -5]. Further studies are needed to scrutinize whether the aetiology of circulatory failure (e.g., sepsis) has a role in this alteration.

### LAS and volume expansion

Our study examined the effect of fluid-induced changes in cardiac preload on LAS. Some evidence exists that LAS may be preload dependent. Previous small studies [20, 22–24] enrolling mainly healthy subjects, have shown an association between preload alteration and LASr. LAS variation during an increase of cardiac preload has only been evaluated by Gottfridsson et al. [24], who documented an increase in LASr during a passive leg raising manoeuvre in healthy young individuals. Our study confirms these findings, as we described a major increase in LASr during rapid fluid administration. The conduit and contraction phases of left atrial function have been poorly studied during controlled load alterations.

There is some evidence [23–25] indicating that LASct is relatively unaffected by preload variations. However, the Valsalva manoeuvre and continuous positive airway pressure application used in studies by Gottfridsson et al. [23, 24] shall not produce significant changes in preload in all subjects. In our study, we found a statistically significant increase in LASr and LASct with fluid loading in responders, along with an increase in LV systolic strain. These finding are consistent with cardiovascular physiology. Mechanistically, LASr is coupled to LV longitudinal shortening since the two chambers are anatomically connected, and in systole, the LV exerts a direct stretching effect on the atrium [5]. In fluid responsive patients, ventricular contractile function improves and this could be an important contributing mechanism to the rise in LASr in this setting. The absence of significant correlation between LAS variation and LV longitudinal strain seems to contradict these assumptions and needs further explorations. Similar to LV, LA pump function is theoretically determined by preload (Frank–Starling mechanism), a fact that may explain our finding of increased LASct with fluid loading in responders. Supporting this hypothesis, we found a tendency for an increase in left atrial ejection fraction during volume expansion in fluid responders, although not statistically significant.

Ünlü et al. [25] found that in patients with end-stage renal disease, LASr and LAScd declined during the haemodialysis session. In our study, both LASr and LAScd increased in non-responders during fluid loading. These observations may suggest the role of LA pressure, but warrant further research, inasmuch as the absolute changes in LAScd values were small in our cohort.

### Fluid intolerance

Left atrial strain has been used in cardiology patients for identifying elevated left ventricular filling pressure [8] and for providing a more accurate categorization of diastolic dysfunction than do conventional echocardiographic variables [40]. Similarly, LA strain measure could be used to detect fluid intolerance in critically ill patients receiving a fluid bolus, outperforming traditional echocardiographic indices of diastolic dysfunction.

The majority of studied patients did not show echocardiographic criteria of diastolic dysfunction according to EACVI criteria [37], neither before nor after fluid administration. More specifically, we found that only one patient could be classified as having elevated left ventricular filling pressure and three patients indetermined LV filling pressure at baseline. The absence of significant correlation between LA strain and conventional indices of diastolic dysfunction is consistent with the characteristics of included patients and timing of echocardiographic examination: we enrolled patients early after the onset of acute circulatory failure and before substantial fluid resuscitation.

In our study we didn’t find any difference in the three components of LAS (LASr, LAScd and LASct) between fluid responders and non-responders patients, nor in conventional echocardiographic criteria of diastolic dysfunction (except for deceleration time of E-wave). Moreover, LASr increased during volume expansion by the same rate in fluid responders and non-responders.

In conclusion, according to our results, LAS doesn’t appear to add clues to the identification of patients that would not benefit from fluid administration, and further studies are warranted to assess its usefulness to detect fluid intolerance.

### Fluid responsiveness

Over the last 20 years, traditional static markers of preload responsiveness, notably central venous pressure [41], have been shown to be unreliable and several dynamic tests have been developed (e.g., pulse pressure variation, stroke volume variation, passive leg raising, end-expiratory occlusion test) [35]. . LAS is assumed to reflect at least in part left ventricular end-diastolic pressure, with lower values of LAS being markers of elevated left ventricular filling pressure [8]. In our study, we showed that a static value of LAS could not detect fluid responsiveness. In this perspective, our finding that LAS components cannot predict fluid responsiveness is consistent with previous literature on static indicators of cardiac preload [36].

### Limitations

Our study has several limitations. First, this is a single-centre study with a cohort of small size; in addition, inclusion rate was slow. Therefore, the generalizability of our results is questionable. Second, we included a general population of critically ill patients needing volume expansion as the clinician acumen and we might have missed the most severe patients; nevertheless, we wanted our study to be pragmatic and thus to describe left atrial strain change during volume expansion in patients that actually receive intravenous resuscitation fluid in clinical practice [42–44]. Unfortunately, we didn’t record the specific trigger for fluid administration in our cohort of patients and we cannot exclude that LAS physiology could be different in specific subgroups. Third, the measurement of LAS requires an experienced operator and the recording of adequate apical views. However, in our study we found a good feasibility (only 5/43 patients were excluded because of inadequate ultrasound imaging) and reproducibility of LAS parameters. Nevertheless, TTE image quality in critically ill patients is often suboptimal and we did not investigate to which extent echogenicity could impact the precision of LAS analysis. Finally, we used change in VTI as a surrogate of increase in stroke volume to define fluid responsiveness. However, echocardiography is widely used nowadays in ICU to track changes in cardiac output and the relative changes in VTI have been validated in this setting [29].

## Conclusion

All components of LAS were severely altered during acute circulatory failure and significantly increased during fluid administration. However, neither their baseline value nor their variation can be used as markers of fluid responsiveness.

### Electronic supplementary material

Below is the link to the electronic supplementary material.

Supplementary Material 1

Supplementary Material 2

Supplementary Material 3

Supplementary Material 4

Supplementary Material 5

Supplementary Material 6

Supplementary Material 7

Supplementary Material 8

## Data Availability

All data generated and analyzed during the study are included in the published article and can be shared upon request. All authors helped to revise the draft of the manuscript. All authors read and approved the final manuscript.

## Abbreviations

ICU: Intensive Care Unit
CO: Cardiac Output
LA: Left Atrium
LV: Left Ventricle
LAS: Left Atrial Strain
AF: Atrial Fibrillation
LASr: Left Atrial Strain reservoir
CPAP: Continuous Positive Airway Pressure
PLR: Passive Leg Raising
LAScd: Left Atrial Strain conduit
LASct: Left Atrial Strain contraction
MAP: Mean Arterial Pressure
HR: Heart Rate
TTE: Trans-Thoracic Echocardiography
LVOT-VTI: Left Ventricular Outflow Tract-Velocity Time Integral
A4C: Apical-four-chamber
A2C: Apical-two-chamber
ROI: Region Of Interest
EACVI: European Association of CadioVascular Imaging
ASE: American Society of Echocardiography
ΔLAS: Change in Left Atrial Strain

## References

[1] Cecconi M, De Backer D, Antonelli M, Beale R, Bakker J, Hofer C, Jaeschke R, Mebazaa A, Pinsky MR, Teboul JL, Vincent JL, Rhodes A (2014). Consensus on circulatory shock and hemodynamic monitoring. Task force of the European Society of Intensive Care Medicine. Intensive Care Med.

[2] Michard F, Teboul JL. Predicting fluid responsiveness in ICU patients: a critical analysis of the evidence. Chest. 2002;121(6):2000-8. 10.1378/chest.121.6.2000. PMID: 12065368. 10.1378/chest.121.6.2000 12065368

[3] Bowcock EM, Mclean A (2022). Bedside assessment of left atrial pressure in critical care: a multifaceted gem. Crit Care.

[4] Thomas L, Marwick TH, Popescu BA, Donal E, Badano LP. Left Atrial Structure and Function, and Left Ventricular Diastolic Dysfunction: JACC State-of-the-Art Review. J Am Coll Cardiol. 2019;73(15):1961–1977. 10.1016/j.jacc.2019.01.059. PMID: 31000000. 10.1016/j.jacc.2019.01.059 31000000

[5] Smiseth OA, Baron T, Marino PN, Marwick TH, Flachskampf FA. Imaging of the left atrium: pathophysiology insights and clinical utility. Eur Heart J Cardiovasc Imaging. 2021;23(1):2–13. 10.1093/ehjci/jeab191. PMID: 34601594. 10.1093/ehjci/jeab191 34601594

[6] Sun BJ, Park JH. Echocardiographic Measurement of Left Atrial Strain - A Key Requirement in Clinical Practice. Circ J. 2021;86(1):6–13. 10.1253/circj.CJ-21-0373. Epub 2021 Jun 5. PMID: 34092759. 10.1253/circj.CJ-21-0373 34092759

[7] Cameli M, Mandoli GE, Loiacono F, Dini FL, Henein M, Mondillo S. Left atrial strain: a new parameter for assessment of left ventricular filling pressure. Heart Fail Rev. 2016;21(1):65–76. 10.1007/s10741-015-9520-9. PMID: 26687372. 10.1007/s10741-015-9520-9 26687372

[8] Inoue K, Khan FH, Remme EW, Ohte N, García-Izquierdo E, Chetrit M, Moñivas-Palomero V, Mingo-Santos S, Andersen ØS, Gude E, Andreassen AK, Wang TKM, Kikuchi S, Stugaard M, Ha JW, Klein AL, Nagueh SF, Smiseth OA (2021). Determinants of left atrial reservoir and pump strain and use of atrial strain for evaluation of left ventricular filling pressure. Eur Heart J Cardiovasc Imaging.

[9] Cameli M, Sparla S, Losito M, Righini FM, Menci D, Lisi M, D’Ascenzi F, Focardi M, Favilli R, Pierli C, Fineschi M, Mondillo S (2016). Correlation of left atrial strain and doppler measurements with Invasive Measurement of Left Ventricular End-Diastolic pressure in patients stratified for different values of Ejection Fraction. Echocardiography.

[10] Zhou Y, Zhao CM, Shen ZY, Zhao X, Zhou BY (2021). Mitral early-diastolic inflow peak velocity (E)-to-left atrial strain ratio as a novel index for predicting elevated left ventricular filling pressures in patients with preserved left ventricular ejection fraction. Cardiovasc Ultrasound.

[11] Lin J, Ma H, Gao L, Wang Y, Wang J, Zhu Z, Pang K, Wang H, Wu W (2020). Left atrial reservoir strain combined with E/E’ as a better single measure to predict elevated LV filling pressures in patients with coronary artery disease. Cardiovasc Ultrasound.

[12] Fan JL, Su B, Zhao X, Zhou BY, Ma CS, Wang HP, Hu SD, Zhou YF, Ju YJ, Wang MH (2020). Correlation of left atrial strain with left ventricular end-diastolic pressure in patients with normal left ventricular ejection fraction. Int J Cardiovasc Imaging.

[13] Sachdeva S, Desai R, Andi K, Vyas A, Deliwala S, Sachdeva R, Kumar G (2021). Reduced left atrial strain can predict stroke in atrial fibrillation - A meta-analysis. Int J Cardiol Heart Vasc.

[14] Hadadi M, Mohseni-Badalabadi R, Hosseinsabet A (2021). Assessment of the ability of the CHA2DS2-VASc scoring system to grade left atrial function by 2D speckle-tracking echocardiography. BMC Cardiovasc Disord.

[15] Koca H, Demirtas AO, Kaypaklı O, Icen YK, Sahin DY, Koca F, Koseoglu Z, Baykan AO, Guler EC, Demirtas D, Koc M (2020). Decreased left atrial global longitudinal strain predicts the risk of atrial fibrillation recurrence after cryoablation in paroxysmal atrial fibrillation. J Interv Card Electrophysiol.

[16] Motoc A, Luchian ML, Scheirlynck E, Roosens B, Chameleva H, Gevers M, Galloo X, von Kemp B, Ramak R, Sieira J, de Asmundis C, Chierchia GB, Magne J, Weytjens C, Droogmans S, Cosyns B (2021). Incremental value of left atrial strain to predict atrial fibrillation recurrence after cryoballoon ablation. PLoS ONE.

[17] Weber J, Bond K, Flanagan J, Passick M, Petillo F, Pollack S, Robinson N, Petrossian G, Cao JJ, Barasch E (2021). The Prognostic Value of Left Atrial Global Longitudinal strain and left atrial phasic volumes in patients undergoing transcatheter valve implantation for severe aortic stenosis. Cardiology.

[18] Cameli M, Pastore MC, Righini FM, Mandoli GE, D’Ascenzi F, Lisi M, Nistor D, Sparla S, Curci V, Di Tommaso C, Marino F, Stricagnoli M, Mondillo S. Prognostic value of left atrial strain in patients with moderate asymptomatic mitral regurgitation. Int J Cardiovasc Imaging. 2019;35(9):1597–1604. 10.1007/s10554-019-01598-6. Epub 2019 Apr 10. PMID: 30972528. 10.1007/s10554-019-01598-6 30972528

[19] Beyls C, Hermida A, Bohbot Y (2021). Automated left atrial strain analysis for predicting atrial fibrillation in severe COVID-19 pneumonia: a prospective study. Ann Intensive Care.

[20] Franchi F, Faltoni A, Cameli M, Muzzi L, Lisi M, Cubattoli L, Cecchini S, Mondillo S, Biagioli B, Taccone FS, Scolletta S (2013). Influence of positive end-expiratory pressure on myocardial strain assessed by speckle tracking echocardiography in mechanically ventilated patients. Biomed Res Int.

[21] Cameli M, Bigio E, Lisi M, Righini FM, Galderisi M, Franchi F, Scolletta S, Mondillo S. Relationship between pulse pressure variation and echocardiographic indices of left ventricular filling pressure in critically ill patients. Clin Physiol Funct Imaging. 2015;35(5):344 – 50. 10.1111/cpf.12168. Epub 2014 Jun 5. PMID: 24902871. 10.1111/cpf.12168 24902871

[22] Genovese D, Singh A, Volpato V, Kruse E, Weinert L, Yamat M, Mor-Avi V, Addetia K, Lang RM (2018). Load dependency of left atrial strain in normal subjects. J Am Soc Echocardiogr.

[23] Gottfridsson P, Law L, A’roch R, Myrberg T, Hultin M, Lindqvist P, Haney M (2023). Left atrial contraction strain during a Valsalva manoeuvre: a study in healthy humans. Clin Physiol Funct Imaging.

[24] Gottfridsson P, A’Roch R, Lindqvist P, Law L, Myrberg T, Hultin M, A’Roch A, Haney M. Left atrial contraction strain and controlled preload alterations, a study in healthy individuals. Cardiovasc Ultrasound. 2022;20(1):8. 10.1186/s12947-022-00278-1. Erratum in: Cardiovasc Ultrasound. 2022;20(1):12. PMID: 35354482; PMCID: PMC8966341. 10.1186/s12947-022-00278-1 PMC8966341 35354482

[25] Ünlü S, Yamak BA, Sezenöz B, Şahinarslan A, Arınsoy ST (2021). Left atrial contractile longitudinal strain determines intrinsic left atrial function regardless of load status and left ventricular deformation. Int J Cardiovasc Imaging.

[26] Park CS, Kim YK, Song HC, Choi EJ, Ihm SH, Kim HY, Youn HJ, Seung KB. Effect of preload on left atrial function: evaluated by tissue Doppler and strain imaging. Eur Heart J Cardiovasc Imaging. 2012;13(11):938 – 47. 10.1093/ehjci/jes069. Epub 2012 Apr 18. PMID: 22514009. 10.1093/ehjci/jes069 22514009

[27] Lancellotti P, Price S, Edvardsen T, Cosyns B, Neskovic AN, Dulgheru R, Flachskampf FA, Hassager C, Pasquet A, Gargani L, Galderisi M, Cardim N, Haugaa KH, Ancion A, Zamorano JL, Donal E, Bueno H, Habib G (2015). The use of echocardiography in acute cardiovascular care: recommendations of the European Association of Cardiovascular Imaging and the Acute Cardiovascular Care Association. Eur Heart J Acute Cardiovasc Care.

[28] Lang RM, Badano LP, Mor-Avi V, Afilalo J, Armstrong A, Ernande L, Flachskampf FA, Foster E, Goldstein SA, Kuznetsova T, Lancellotti P, Muraru D, Picard MH, Rietzschel ER, Rudski L, Spencer KT, Tsang W, Voigt JU. Recommendations for cardiac chamber quantification by echocardiography in adults: an update from the American Society of Echocardiography and the European Association of Cardiovascular Imaging. J Am Soc Echocardiogr. 2015;28(1):1–39.e14. 10.1016/j.echo.2014.10.003. PMID: 25559473. 10.1016/j.echo.2014.10.003 25559473

[29] Jozwiak M, Mercado P, Teboul JL (2019). What is the lowest change in cardiac output that transthoracic echocardiography can detect?. Crit Care.

[30] Badano LP, Kolias TJ, Muraru D, Abraham TP, Aurigemma G, Edvardsen T, D’Hooge J, Donal E, Fraser AG, Marwick T, Mertens L, Popescu BA, Sengupta PP, Lancellotti P, Thomas JD, Voigt JU. Industry representatives; Reviewers: This document was reviewed by members of the 2016–2018 EACVI Scientific Documents Committee. Standardization of left atrial, right ventricular, and right atrial deformation imaging using two-dimensional speckle tracking echocardiography: a consensus document of the EACVI/ASE/Industry Task Force to standardize deformation imaging. Eur Heart J Cardiovasc Imaging. 2018;19(6):591–600. 10.1093/ehjci/jey042. Erratum in: Eur Heart J Cardiovasc Imaging. 2018;19(7):830–833. PMID: 29596561. 10.1093/ehjci/jey042 29596561

[31] Donal E, Galli E, Schnell F (2017). Left atrial strain: a must or a plus for routine clinical practice?. Circ Cardiovasc Imaging.

[32] Donal E, Behagel A, Feneon D (2015). Value of left atrial strain: a highly promising field of investigation. Eur Heart J Cardiovasc Imaging.

[33] Gan GCH, Ferkh A, Boyd A, Thomas L (2018). Left atrial function: evaluation by strain analysis. Cardiovasc Diagn Ther.

[34] Vincent JL, Weil MH. Fluid challenge revisited. Crit Care Med. 2006;34(5):1333-7. 10.1097/01.CCM.0000214677.76535.A5. PMID: 16557164. 10.1097/01.CCM.0000214677.76535.A5 16557164

[35] Monnet X, Marik PE, Teboul JL (2016). Prediction of fluid responsiveness: an update. Ann Intensive Care.

[36] Monnet X, Teboul JL. Assessment of fluid responsiveness: recent advances. Curr Opin Crit Care. 2018;24(3):190–195. 10.1097/MCC.0000000000000501. PMID: 29634494. 10.1097/MCC.0000000000000501 29634494

[37] Nagueh SF, Smiseth OA, Appleton CP, Byrd BF 3rd, Dokainish H, Edvardsen T, Flachskampf FA, Gillebert TC, Klein AL, Lancellotti P, Marino P, Oh JK, Popescu BA, Waggoner AD. Recommendations for the Evaluation of Left Ventricular Diastolic Function by Echocardiography: An Update from the American Society of Echocardiography and the European Association of Cardiovascular Imaging. J Am Soc Echocardiogr. 2016;29(4):277–314. 10.1016/j.echo.2016.01.011. PMID: 27037982. 10.1016/j.echo.2016.01.011 27037982

[38] Pathan F, D’Elia N, Nolan MT, Marwick TH, Negishi K (2017). Normal ranges of left atrial strain by Speckle-Tracking Echocardiography: a systematic review and Meta-analysis. J Am Soc Echocardiogr.

[39] Tadafumi Sugimoto S, Robinet R, Dulgheru (2018). Echocardiographic reference ranges for normal left atrial function parameters: results from the EACVI NORRE study. Eur Heart J - Cardiovasc Imaging.

[40] Nagueh SF, Khan SU. Left Atrial Strain for Assessment of Left Ventricular Diastolic Function: Focus on Populations With Normal LVEF. JACC Cardiovasc Imaging. 2023;16(5):691–707. 10.1016/j.jcmg.2022.10.011. Epub 2023 Jan 11. PMID: 36752445. 10.1016/j.jcmg.2022.10.011 36752445

[41] Hamzaoui O, Gouëzel C, Jozwiak M, Millereux M, Sztrymf B, Prat D, Jacobs F, Monnet X, Trouiller P, Teboul JL. Increase in Central Venous Pressure During Passive Leg Raising Cannot Detect Preload Unresponsiveness. Crit Care Med. 2020;48(8):e684-e689. doi: 10.1097/CCM.0000000000004414. Erratum in: Crit Care Med. 2021;49(6):e662. PMID: 32697509. 10.1097/CCM.0000000000004414 32697509

[42] Hammond NE, Taylor C, Finfer S, Machado FR, An Y, Billot L, Bloos F, Bozza F, Cavalcanti AB, Correa M, Du B, Hjortrup PB, Li Y, McIntryre L, Saxena M, Schortgen F, Watts NR, Myburgh J, Fluid, The ANZICS Clinical Trials Group (2017).

[43] Boulain T, Boisrame-Helms J, Ehrmann S, Lascarrou JB, Bouglé A, Chiche A, Lakhal K, Gaudry S, Perbet S, Desachy A, Cabasson S, Geneau I, Courouble P, Clavieras N, Massanet PL, Bellec F, Falquet Y, Réminiac F, Vignon P, Dequin PF, Meziani F (2015). Volume expansion in the first 4 days of shock: a prospective multicentre study in 19 French intensive care units. Intensive Care Med.

[44] Cecconi M, Hofer C, Teboul JL, Pettila V, Wilkman E, Molnar Z, Della Rocca G, Aldecoa C, Artigas A, Jog S, Sander M, Spies C, Lefrant JY, De Backer D, FENICE Investigators; ESICM Trial Group. Fluid challenges in intensive care: the FENICE study: A global inception cohort study. Intensive Care Med. 2015;41(9):1529-37. 10.1007/s00134-015-3850-x. Epub 2015 Jul 11. Erratum in: Intensive Care Med. 2015;41(9):1737-8. multiple investigator names added. PMID: 26162676; PMCID: PMC4550653. 10.1007/s00134-015-3850-x PMC4550653 26162676

