# Supplementary figures and images for: Effect of volume infusion on left atrial strain in acute circulatory failure
Source: Ann Intensive Care. 2024 Apr 9;14:53. doi: 10.1186/s13613-024-01274-6 (PMC11003961; doi:10.1186/s13613-024-01274-6)

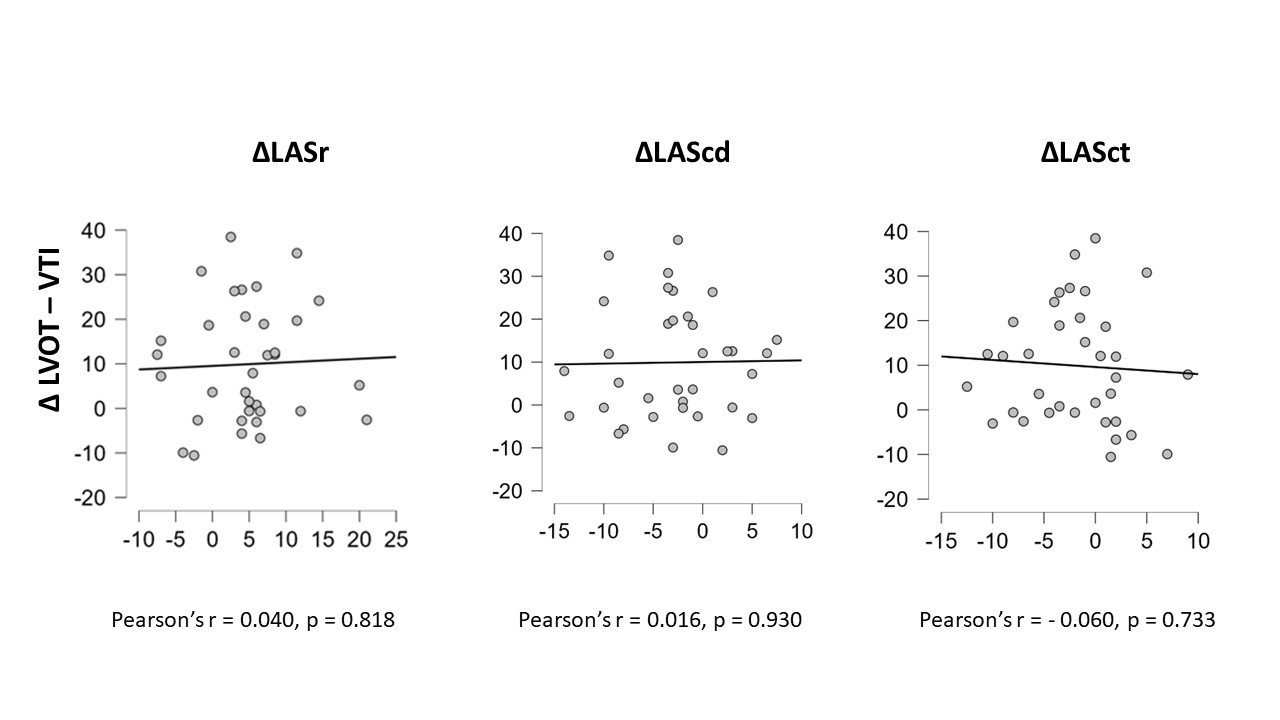

Supplement: Supplementary file 2 — Supplementary Material 2 [file 13613_2024_1274_MOESM2_ESM.png]

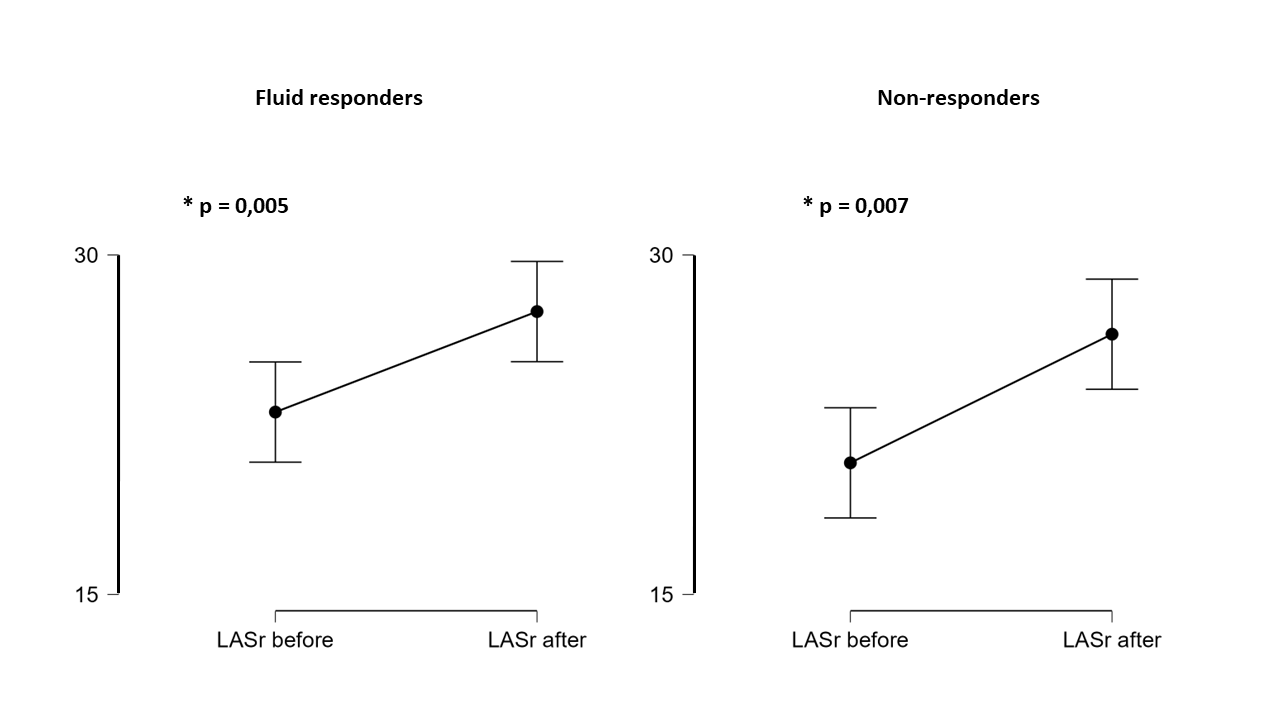

Supplement: Supplementary file 3 — Supplementary Material 3 [file 13613_2024_1274_MOESM3_ESM.png]

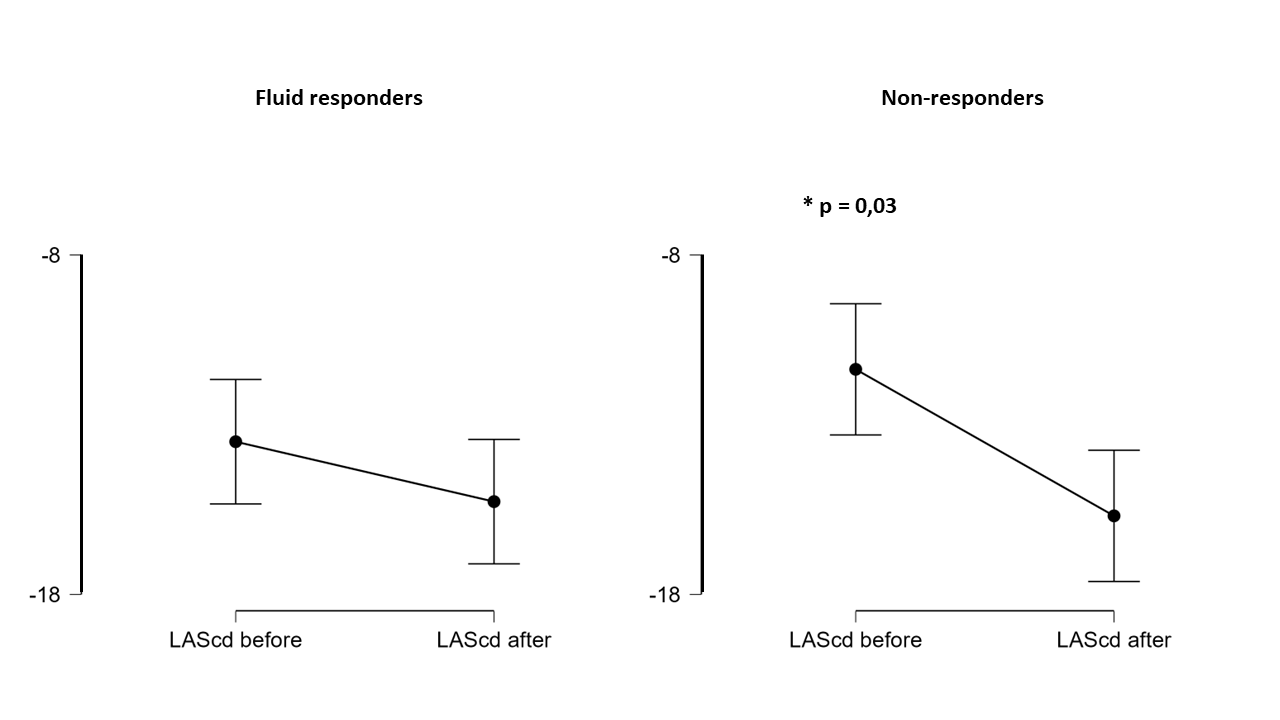

Supplement: Supplementary file 4 — Supplementary Material 4 [file 13613_2024_1274_MOESM4_ESM.png]

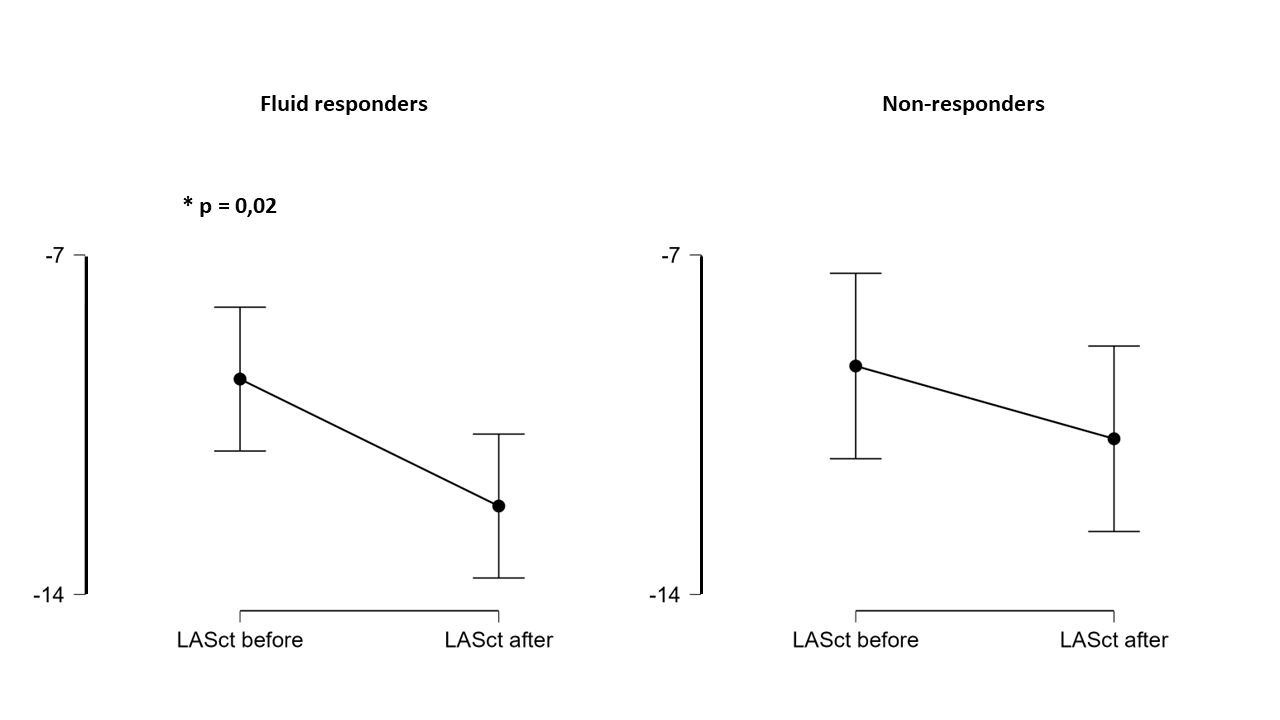

Supplement: Supplementary file 5 — Supplementary Material 5 [file 13613_2024_1274_MOESM5_ESM.png]

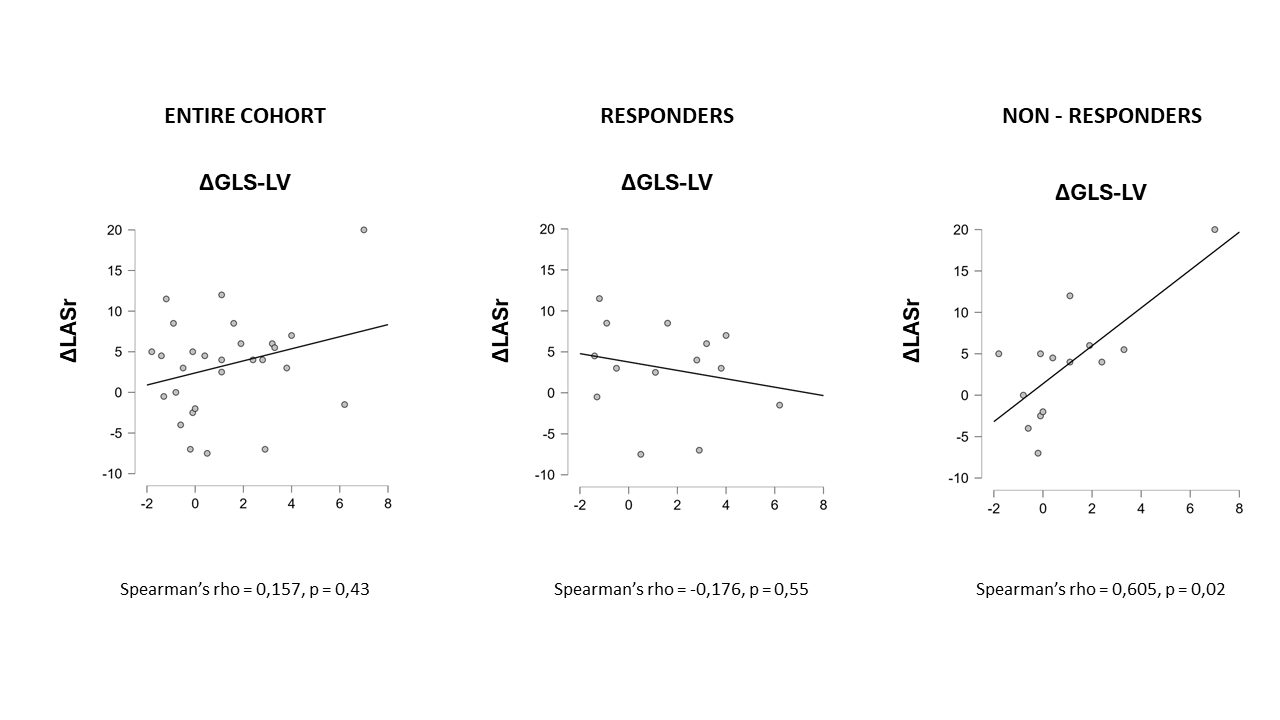

Supplement: Supplementary file 6 — Supplementary Material 6 [file 13613_2024_1274_MOESM6_ESM.png]

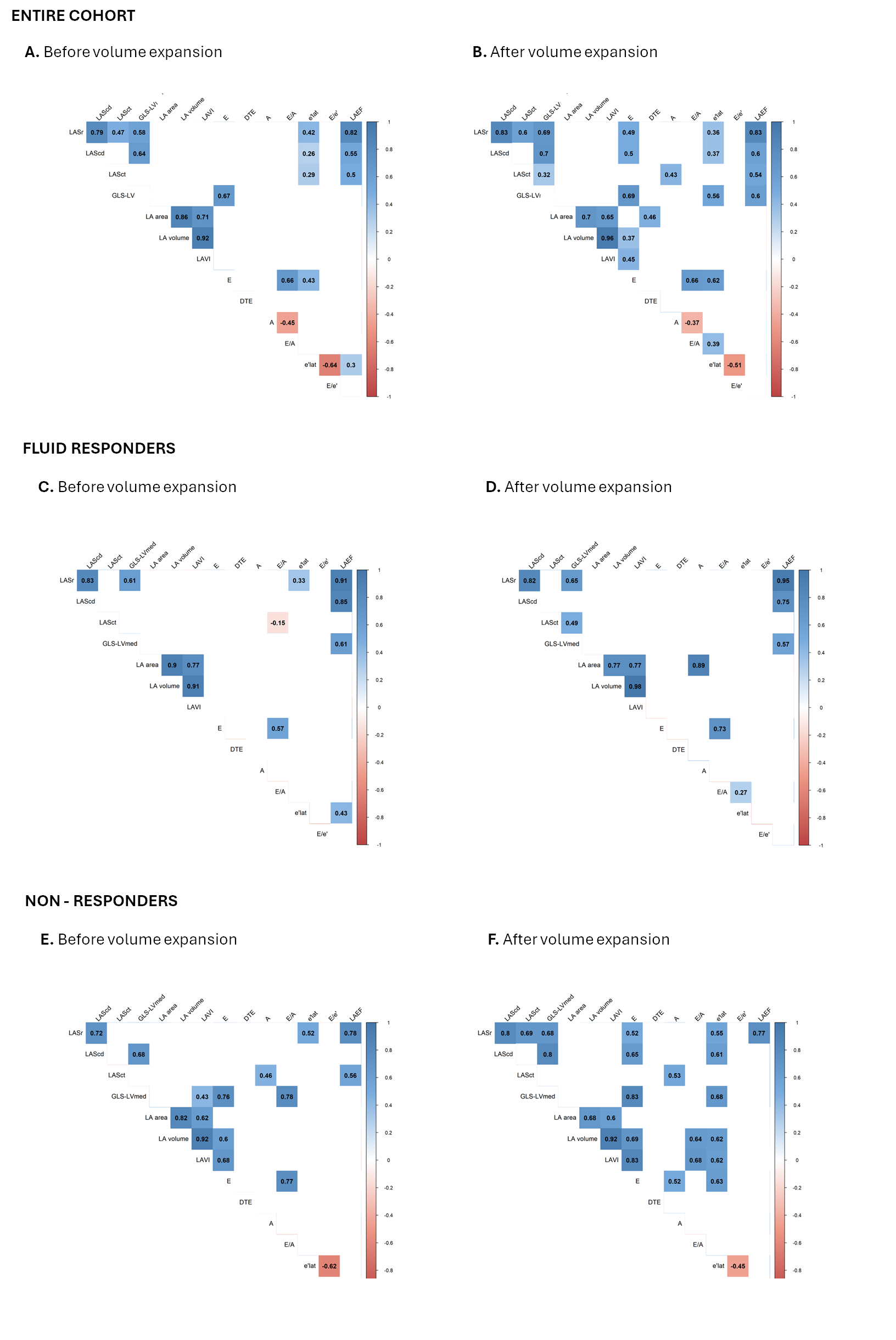

Supplement: Supplementary file 7 — Supplementary Material 7 [file 13613_2024_1274_MOESM7_ESM.png]
