# Supplementary material for: Effect of volume infusion on left atrial strain in acute circulatory failure
Source: Ann Intensive Care. 2024 Apr 9;14:53. doi: 10.1186/s13613-024-01274-6 (PMC11003961; doi:10.1186/s13613-024-01274-6)
Supplement: Supplementary file 8 — Supplementary Material 8 [file 13613_2024_1274_MOESM8_ESM.docx]

| **Table S1. Reproducibility of left atrial strain analysis** | | | | | |
| --- | --- | --- | --- | --- | --- |
| LA strain parameters | ICC for inter‐observer variability | | 95% CI | ICC for intra-observer variability | 95% CI |
| LASr mean | | 0.88 | 0.76-0.94 | 0.92 | 0.68-0.98 |
| LAScd mean | | 0.88 | 0.77-0.94 | 0.81 | 0.38-0.95 |
| LASct mean | | 0.84 | 0.69-0.92 | 0.95 | 0.81-0.99 |
| * ICC indicates intraclass correlation coefficient; CI, confidence interval; LA, left atrial; LASr left atrial strain reservoir; LAScd, left atrial strain conduit; LASct left atrial strain contraction. | | | | | |
